# Enhancing oil production in *Arabidopsis* through expression of a ketoacyl-ACP synthase domain of the PUFA synthase from *Thraustochytrium*

**DOI:** 10.1186/s13068-019-1514-8

**Published:** 2019-06-29

**Authors:** Xi Xie, Dauenpen Meesapyodsuk, Xiao Qiu

**Affiliations:** Department of Food and Bioproduct Sciences, University of Saskatchewan, Saskatoon, SK S7N 5A8 Canada

**Keywords:** PUFA synthase, KS domain, Oil content, Seed weight, *Thraustochytrium*

## Abstract

**Background:**

Plant seed oil is an important bioresource for human food and animal feed, as well as industrial bioproducts. Therefore, increasing oil content in seeds has been one of the primary targets in the breeding programs of oilseed crops. *Thraustochytrium* is a marine protist that can produce a high level of very long-chain polyunsaturated fatty acids (VLCPUFAs) using a PUFA synthase, a polyketide synthase-like fatty acid synthase with multiple catalytic domains. Our previous study showed that a KS domain from the synthase could complement an *Escherichia coli* mutant defective in β-ketoacyl-ACP synthase I (*FabB*) and increase the total fatty acid production. In this study, this KS domain from the PUFA synthase was further functionally analyzed in *Arabidopsis thaliana* for the capacity of oil production.

**Results:**

The plastidial expression of the KS domain could complement the defective phenotypes of a *KASI* knockout mutant generated by CRISPR/Cas9. Seed-specific expression of the domain in wild-type *Arabidopsis* significantly increased seed weight and seed oil, and altered the unsaturation level of fatty acids in seeds, as well as promoted seed germination and early seedling growth.

**Conclusions:**

The condensation process of fatty acid biosynthesis in plants is a limiting step, and overexpression of the KS domain from a PUFA synthase of microbial origin offers a new strategy to increase oil production in oilseed plants.

**Electronic supplementary material:**

The online version of this article (10.1186/s13068-019-1514-8) contains supplementary material, which is available to authorized users.

## Background

Plant seed oil is an important bioresource not only for food and feed, but also for industrial bioproducts such as biofuel and biopolymers potential for replacing non-renewable petro-polymers and petro-fuels [1–3]. Therefore, increasing oil content in seeds has long been one of the primary targets in the breeding programs of oilseed crops. In the past few years, the success has been made through various molecular breeding approaches, such as manipulation of transcriptional factors in carbon partitioning, regulation of the biosynthetic process of lipids, and overexpression of key enzymes in fatty acid biosynthesis and assembly. For instance, overproduction of the regulator WRINKLED1 in *Arabidopsis thaliana* resulted in enlarged seeds and 10–40% oil increase [4]. Overexpression of a yeast glycerol-3-phosphate dehydrogenase for the formation of glycerol-3-phosphate at the starting point of the Kennedy pathway boosted oil content by 40% in the seeds of oilseed rape [5]. Seed-specific expression of acyl-CoA:diacylglycerol transferase 1 (DGAT1) for the biosynthesis of triacylglycerol (TAG) enhanced oil content by about 20% in *A. thaliana* [6]. In addition, increased activity of plastidial enzymes such as biotin carboxyl carrier protein isoform 2 (BCCP2) in acetyl-coenzyme carboxylase (ACCase), pyruvate transporter (BASS2) and purple acid phosphatase 2 could also raise oil levels in oilseeds [7–9].

In plant, fatty acid biosynthesis occurs in plastids where acetyl-CoA carboxylase (ACCase) catalyzes the formation of malonyl-CoA from acetyl-CoA initially, and then, a type II fatty acid synthase (FAS) complex uses malonyl thioester as an extender for the synthesis of long-chain saturated fatty acids such as 16:0 and 18:0 through a repetitive cycle of four catalytic reactions: condensation, keto-reduction, dehydration and enoyl-reduction [10]. After synthesis, these two long-chain fatty acids are often desaturated by a soluble acyl-ACP Δ9-desaturase in the stroma of plastids giving 16:1–9 and 18:1–9. Subsequently, these saturated and monounsaturated long-chain fatty acids are exported to cytosol where they can be further modified by desaturations to introduce more double bonds or elongations to extend the chain length. With few exceptions [11], higher plants do not possess capacity to synthesize very long-chain polyunsaturated fatty acids (VLCPUFAs) with the chain length of 20C or more and the double bonds of two or more.

De novo biosynthesis of VLCPUFAs occurs mainly in microorganisms where their biosynthesis goes through either an aerobic pathway catalyzed by desaturases and elongases or an anaerobic pathway catalyzed by a polyunsaturated fatty acid (PUFA) synthase. *Thraustochytrium* is a protist producing a high level of a nutritionally important VLCPUFA docosahexaenoic acid (DHA, 22:6-n3). Biosynthesis of this fatty acid is catalyzed by a PUFA synthase, a polyketide synthase-like mega-enzyme [12, 13]. Structurally, the PUFA synthase shares similarity to both type I and type II fatty acid synthases in catalytic domains and motifs. Functionally, both PUFA synthase, and type I and II fatty acid synthases catalyze the synthesis of fatty acids through similar reiterative reactions such as condensation, keto-reduction, dehydration and enoyl-reduction using acyl-ACPs as substrates [14–16]. The PUFA synthase in *Thraustochytrium* comprises three large subunits, each with multiple catalytic domains. However, the exact functions of these domains remain poorly understood. Our previous study showed ketoacyl-ACP synthase (KS) domains from the PUFA synthase could functionally complement the defective phenotype of two *E. coli* mutants of β-ketoacyl-ACP synthases. Particularly, overexpression of one KS domain from subunit-B (KS-B) resulted in accumulation of a high level of total fatty acids as well as unsaturated fatty acids in *E. coli* [17]. The aim of this study was to extend our previous research on this domain to model oilseed plant *Arabidopsis* for further functional analysis. The results showed that the KS domain was functional in the plant and able to complement the defective phenotype of an *Arabidopsis KASI* knockout mutant generated by clustered regularly interspaced short palindromic repeats/CRISPR-associated  protein-9 nuclease (CRISPR/Cas9), and the seed-specific expression of this domain in wild-type *Arabidopsis* significantly enhanced seed weight and seed oil and promoted seed germination and seedling growth. These results emphasize that the condensation process of the fatty acid biosynthesis in plants is a limiting step and the enhanced activity by overexpression of the KS domain from a PUFA synthase of microbial origin offers an attractive biotechnological approach to increase oil content in oilseed plants.

## Results

### Sequence comparison of the KS domain with β-ketoacyl-ACP synthase I from plants

**Fig. 1:**
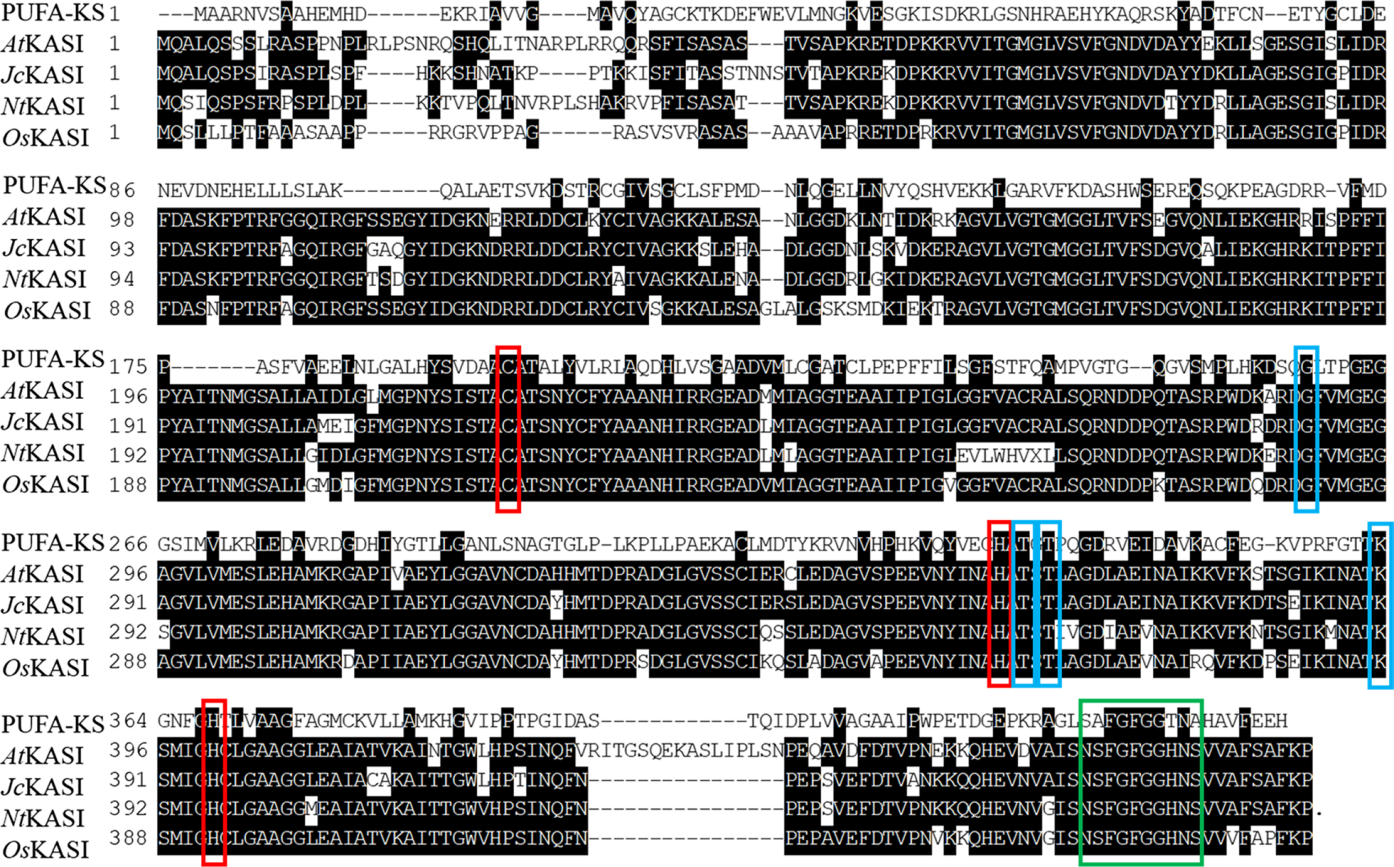
Comparison of the KS domain with KASI from plants. Red boxes represent conserved residues directly involved in the catalysis. Blue boxes represent conserved residues interacting with substrate, and the green box represents a glycine motif in the C-terminus. PUFA-KS, PUFA synthase (PRJNA368972); *At*, *Arabidopsis thaliana* (NM_001036941); *Jc*, *Jatropha curcas L*. (KDP37953) *Nt*, *Nicotiana tabacum* (KX033513); *Os*, *Oryza sativa* (LOC_Os06g09630)

The PUFA synthase from *Thraustochytrium* was comprised of three subunits encoded by three large open reading frames (ORFs), each with multiple catalytic domains predicted by the presence of characteristic active site sequence motifs or residues [13]. One KS domain (KS-B) from residue 1 to 469 was identified in subunit-B of the PUFA synthase. Comparison of this KS domain with known plant β-ketoacyl-ACP synthase I (KASI) (Fig. 1) showed that it shared only about 20% amino acid sequence identity to plant KAS I from *A. thaliana*, *Jatropha curcas*, *Nicotiana tabacum*, *Oryza sativa* throughout the entire sequence. However, key residues in the active site such as a cysteine (C), two histidines (H) and a lysine (K) essential for the decarboxylation during the condensation process were completely conserved among these sequences. In addition, a glycine (G) residue presumably located at the accepting entrance of the substrate-binding tunnel and two threonine (T) residues likely involved in hydrogen bonding with the ACP phosphopantetheine moiety were also highly conserved. Furthermore, a glycine-rich motif located in the C-terminus contributing to the formation of an oxyanion hole for decarboxylation reaction was also observed among these sequences [18–20].

### Generation of the constructs disrupting endogenous *KASI* and expressing the KS domain in *Arabidopsis*

**Fig. 2:**
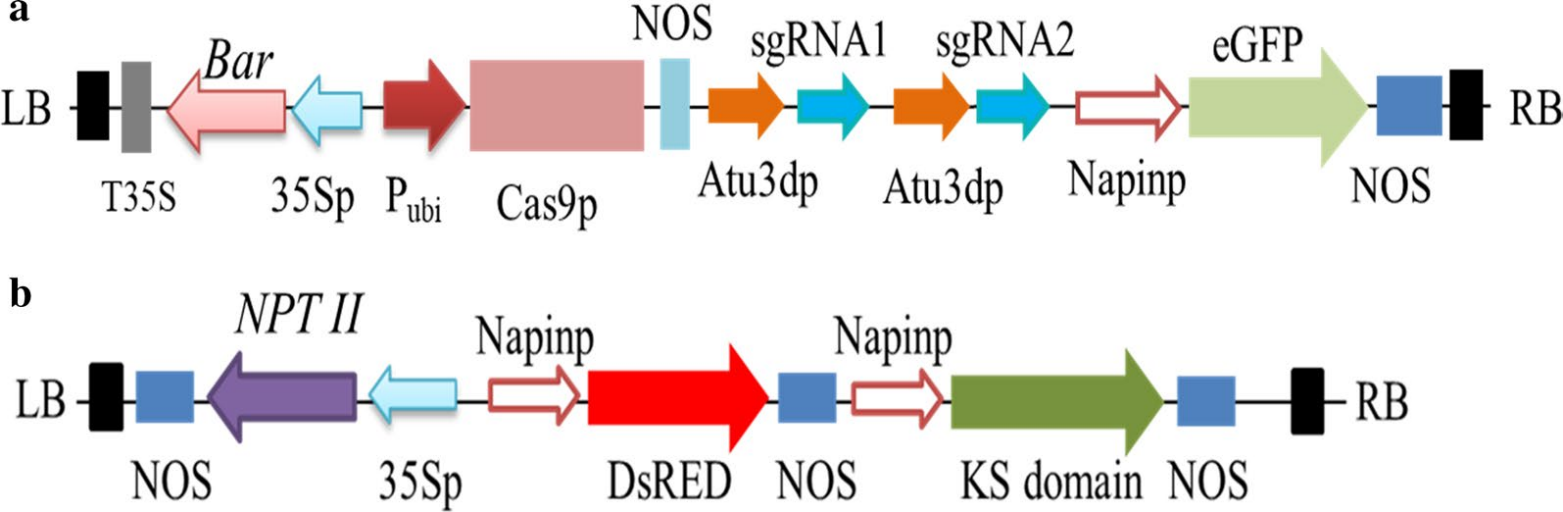
Schematic representation of the T-DNA region of two binary vectors. **a** The T-DNA for of *AtKASI*-disrupting plasmid. **b** The T-DNA region of the KS domain expression plasmid

To functionally analyze the KS domain in *Arabidopsis*, two binary plasmids were constructed. The first one was used to disrupt endogenous *KASI* genes, and the other was used to express the KS domain as a standalone enzyme from the PUFA synthase. To generate a construct to disrupt the *KASI* gene in *Arabidopsis*, two sets of overlapping sgRNA primer sequences targeting the conserved coding region of the gene were synthesized. The adaptor formed from each set of primers with a sgRNA sequence was inserted behind an *Arabidopsis* U3 promoter in an intermediate plasmid pLYsgRNA-AtU3d, giving a sgRNA expression cassette. Two sgRNA expression cassettes were amplified with universal primer sets [21] and assembled into a CRISPR/Cas9 binary vector pYLCRISPR/Cas9-P_ubi_-B by a single golden gate ligation, giving the *KASI*-disrupting plasmid. Besides the two sgRNA expression cassettes for guiding the disruption of targeted genes, this construct was also comprised of a cassette expressing *Cas9* under a constitutive ubiquitin promoter for cutting the targeted sites, a cassette expressing a herbicide resistant *Bar* gene under a constitutive 35S promoter for selecting transgenic plants, and a cassette expressing a modified green fluorescent protein (eGFP) from *Aequorea victoria* under a seed-specific napin promoter for screening transgenic seeds (Fig. 2a).

The second binary plasmid was constructed to express the KS domain in *Arabidopsis*. It was comprised of three expression cassettes. The first cassette was to express the KS domain as a standalone protein with a chloroplast transit peptide from *Arabidopsis* fused to the N-terminus under the control of the seed-specific promoter. The second cassette was to express a kanamycin-resistance gene under the control of a constitutive promoter for selecting transgenic plants. The third cassette was to express a red fluorescent protein gene (*DsRed2*) from *Discosoma* under the seed-specific promoter for screening transgenic seeds (Fig. 2b).

After the confirmation of the structures of the two constructs by sequencing, they were introduced into *Arabidopsis* by floral dipping through an *Agrobacterium tumefaciens*-mediated transformation approach. Transgenic seeds with fluorescence were selected under a fluorescent microscope and grown to the next generation for further analysis.

### Complementation of *Arabidopsis kasI* mutant by the KS domain

KASI is essential for the biosynthesis of fatty acids in plant, and thus, knockout mutants of the gene for this enzyme in *Arabidopsis* [22] might not be easily obtained for the functional complementation test. Therefore, for the complementation assays of the KS domain in *Arabidopsis*, two approaches were attempted. The first approach was to generate a *kasI* mutant *Arabidopsis* using a CRISPR/Cas9 technique, and then express the domain in the mutant, if it was amenable. The second approach was to express the domain first in wild-type *Arabidopsis* and then to disrupt the *KASI* by the CRISPR/Cas9 technique.

**Fig. 3:**
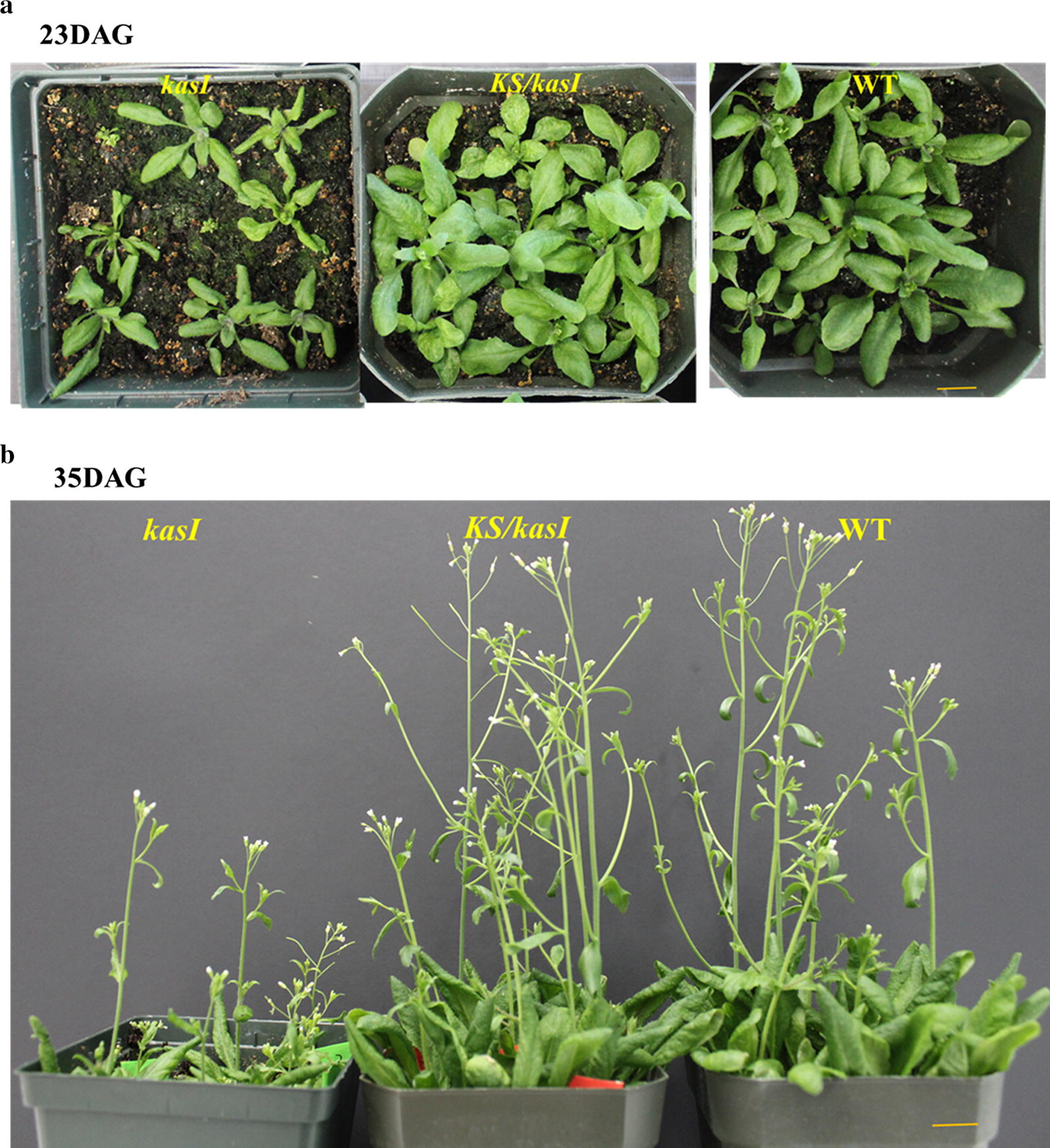
Complementation of the *kasI* mutant in *Arabidopsis*. **a** Growth phenotypes of *kasI* mutant line, *kasI* complementation line (*KS*/*kasI*) at 23 days after growing (DAG). Bars = 2 cm. **b** Growth phenotypes of *kasI* mutant line, *kasI* complementation line (*KS*/*kasI*) at 35 DAG. Bars = 2 cm

To knock out *KASI* in *Arabidopsis*, the disrupting construct was first used to transform wild-type *A. thaliana*. The transgenic seeds were selected under a fluorescent microscope and grown to the next generation for genotyping and phenotyping. Genotyping transgenic T2 plants by sequencing the amplicons of targeted genes identified three homozygous lines with deletion mutation between two targeted sites of the *KASI* gene (*kasI*-1, *kasI*-4 and *kasI*-11). All these lines exhibited similar abnormal growth: smaller and shorter seedlings at the early growth stage and semi-dwarf at the late growth stage (Fig. 3), compared to the wild type. The similar phenotype was previously observed in a *kasI* mutant generated by a T-DNA insertion [22]. Due to the severe growth defect, re-transformation of the mutant with the KS-expression construct for the complementation test was not successful.

As the first approach for the complementation assay was unsuccessful, we then resorted the second approach by expressing the KS domain in *Arabidopsis* first and then disrupting *KASI* under the KS-expressed background. A transgenic plant expressing the KS domain obtained using the second construct for the first transformation was re-transformed with the disrupting construct of CRISPR/Cas9, and transgenic seeds were selected on both green (by eGFP) and red (by *Ds*Red) fluorescence signals and grown to the next generation for genotyping and phenotyping. Genotyping analysis identified two mutation lines (*KS*/*kasI*-9, *KS*/*kasI*-15) with insertion mutation in the target gene. One had a single nucleotide insertion, while the other had a two-nucleotide insertion close to the targeted sites; both resulted in open reading frame-shifting mutations. Phenotypic observations showed that the two transgenic plants had comparable growth and development to the wild type, and no abnormal phenotypes were detected under the growth condition (Fig. 3).

**Fig. 4:**
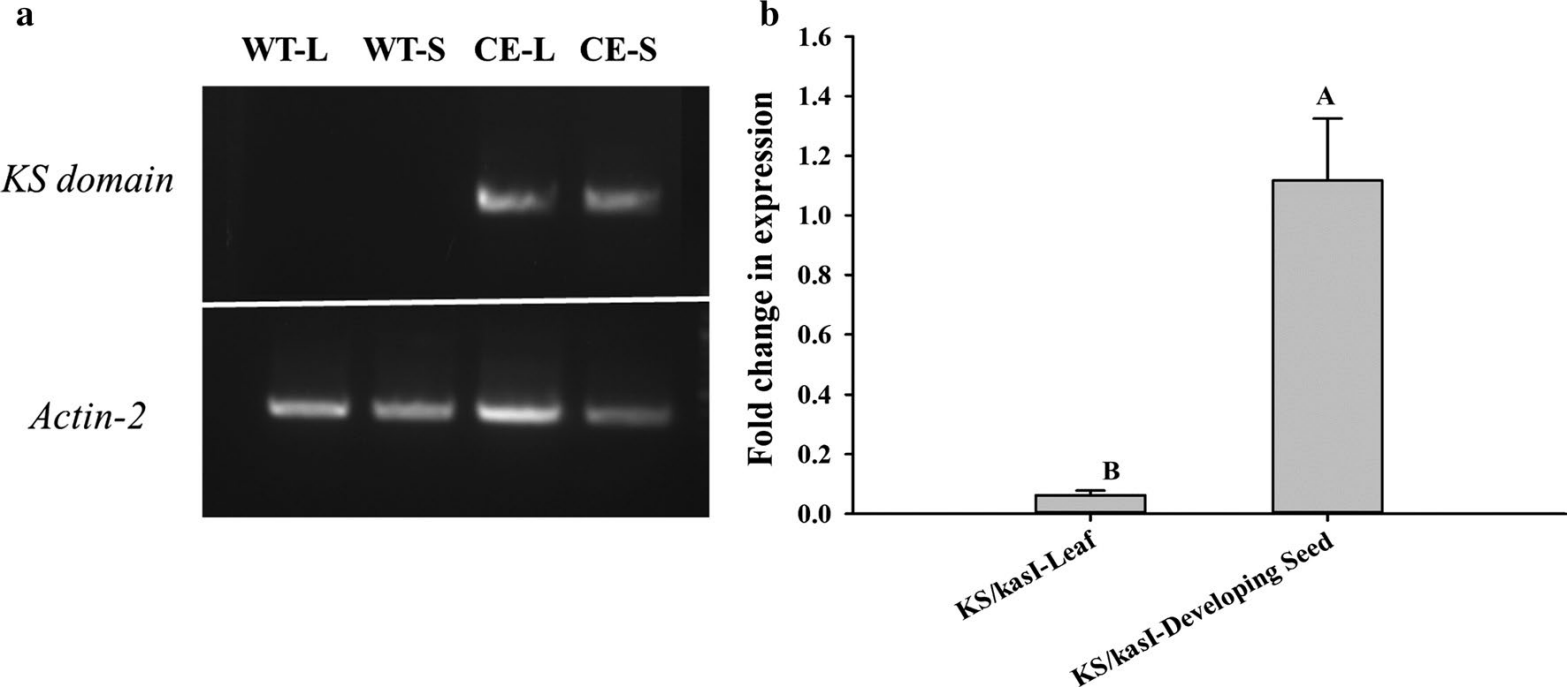
Transcriptional analysis of the *KS* domain in leaves and developing siliques of a complementation line. **a** Expression of the *KS* domain and *AtActin*-*2* in leaves and developing siliques of *KS*/*kasI*-9 analyzed by semi-quantitative RT-PCR. **b** Expression of the *KS* domain in leaves and developing siliques of *KS*/*kasI*-9 analyzed by quantitative RT-PCR. *KS*/*kasI kasI* mutant complementation line, *WT-L* leaves of wild type, *WT-S* siliques of wild type, *CE-L* leaves of complementation line, *CE-S* siliques of complementation line. Values are reported as the means of three biological replicates along with standard deviation. The means with the same letters are not statistically significantly different. Statistical analysis of the results was conducted using the one-way analysis of variance (*P* < 0.05)

To confirm the expression of the KS domain in a complementation line, reverse transcription PCR (RT-PCR) was performed using total RNAs isolated from 7th to 8th rosette leaves and developing siliques at 14 days after flowering [23]. A semi-quantitative PCR analysis showed that no expression of the KS domain was observed in the untransformed *Arabidopsis* control, but the expression of the KS domain could be obviously detected in both leaves and developing siliques of the complementation line (Fig. 4a). A quantitative PCR analysis showed that the transcription level of the KS domain in developing siliques was tenfold higher than that in leaves (Fig. 4b). This result indicated that the KS domain guided by a seed-specific napin promoter indeed possessed significantly higher expression in developing seeds than leaves, but it did express in leaves, providing the function for complementing the *KASI* defective phenotype in *Arabidopsis*.

### Seed-specific expression of the KS domain in wild-type *Arabidopsis*

**Fig. 5:**
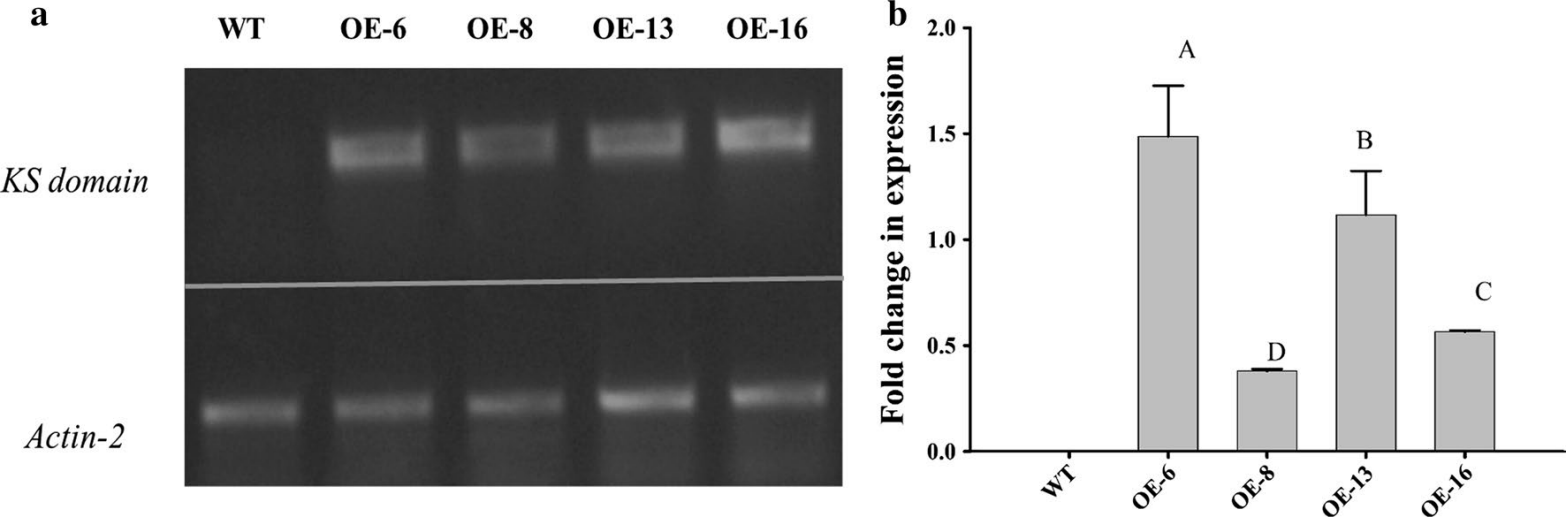
Transcriptional analysis of the *KS* domain in developing siliques of overexpression lines. **a** Expression of the *KS* domain and *AtActin*-*2* in the developing siliques of *KS*/*WT* analyzed by semi-quantitative RT-PCR. **b** Expression of the *KS* domain in leaves and developing siliques of *KS*/*WT* analyzed by quantitative RT-PCR. *WT* wild type, *OE* overexpression lines. Values are reported as the means of three biological replicates along with standard deviation. The means with the same letters are not statistically significantly different. Statistical analysis of the results was conducted using the one-way analysis of variance (*P* < 0.05)

**Fig. 6:**
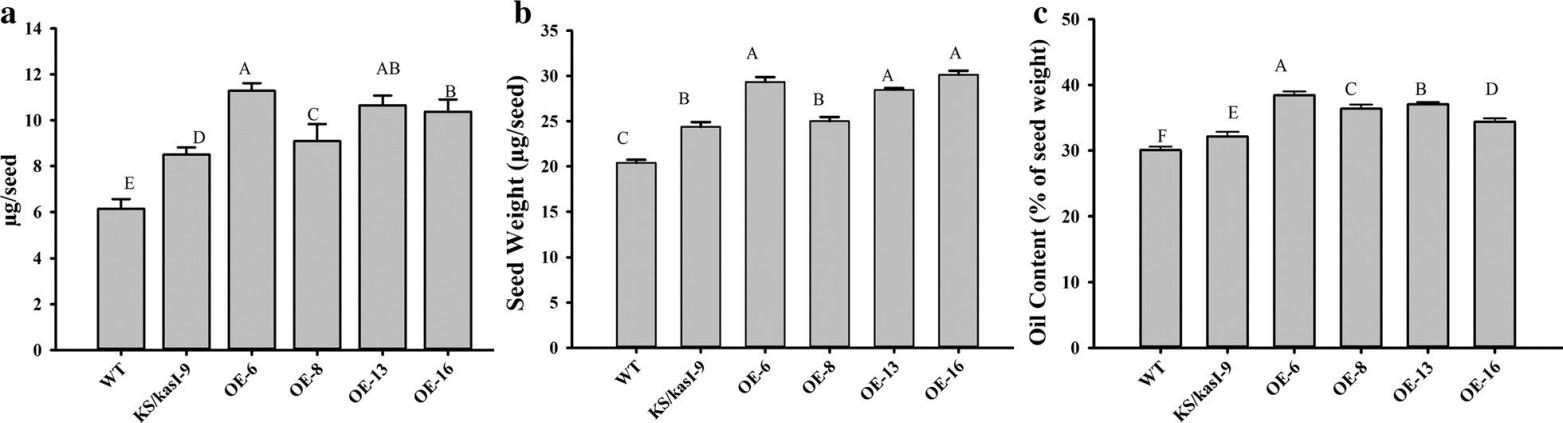
The amount of oil, seed weight and oil content in transgenic seeds overexpressing the KS domain. **a** The amount of oil per seed in the T2 seeds of overexpression lines; **b** the seed weight of the transgenic lines; **c** seed oil content of the transgenic lines. *WT* wild type, *KS*/*kasI kasI* mutant complementation line, *OE* overexpression lines. Values are reported as the means of 10 biological replicates along with standard deviation. The means with the same letters are not statistically significantly different. Statistical analysis of the results was conducted using the one-way analysis of variance (*P* < 0.05)

To examine the impact of the KS domain in fatty acid biosynthesis and oil accumulation in *Arabidopsis*, the second construct expressing the domain under the seed-specific promoter was introduced into wild-type *Arabidopsis*. Transgenic seeds were selected on a red fluorescence signal and propagated to the next generation. To confirm the expression of the KS domain, the total RNAs were isolated from the developing siliques of four transgenic lines (OE-6, OE-8, OE-13 and OE-16) and reverse-transcribed to cDNAs. A semi-quantitative PCR analysis of the cDNAs showed that the expression of the KS domain was much higher than that of a housekeeping gene *Actin*-*2* (Fig. 5a). A quantitative PCR analysis showed that the transcription levels of the KS domain were highly varied among the four lines. The expression level of the KS domain in OE-6 was the highest, followed by OE-16 and OE-13, and OE-8 had the least expression (Fig. 5b). Subsequently, the oil and mass of the seeds in these four transgenic lines were measured. As shown in Fig. 6, all the transgenic lines accumulated more oil relative to the wild-type control and the amount of oil in the mutant complementation line lied between those in the control and transgenic lines. The amount of oil per seed in OE-6, OE-16, OE-13 and OE-8 was increased by 80%, 77%, 69% and 48%, respectively, over the control. In particular, the level of monounsaturated fatty acids (MUFAs) such as C18:1 and C20:1 was significantly higher in these transgenic lines over the wild type, the indicator of the seed triacylglycerol production seen previously [24]. In addition, the decrease in total saturated fatty acids and the increase in total unsaturated fatty acids were also observed in the transgenic lines (Table 1). Intriguingly, the transgenic seeds were also significantly heavier, representing 1.4, 1.2, 1.4 and 1.5-fold increase, respectively, relative to the wild type. Percentages of oil content in these transgenic lines exhibited increases by 14% to 28% over the control. Nevertheless, no significant difference in the seed protein content was detected between these transgenic lines and the control (Additional file 1: Figure S2). These results indicate that expressing the KS domain can significantly increase both seed oil and seed mass in *Arabidopsis*.

**Table 1:** Fatty acid composition (mol%) in transgenic seeds overexpressing the KS domain

FA species	WT	*KS*/*kasI*-9	OE-6	OE-8	OE-13	OE-16
16:0	12.05 ± 0.83*	11.19 ± 0.59*	10.07 ± 0.20	11.72 ± 0.26*	10.27 ± 0.39	10.13 ± 0.23
16:1–9	1.66 ± 0.20	1.37 ± 0.17	0.74 ± 0.03	0.80 ± 0.06	0.66 ± 0.05	2.11 ± 0.18*
18:0	10.73 ± 1.32*	8.84 ± 0.67	7.29 ± 0.38	9.68 ± 0.37*	8.38 ± 0.33	7.71 ± 0.52
18:1–9	11.19 ± 0.82	13.77 ± 0.86	17.38 ± 0.70*	11.98 ± 0.77	17.93 ± 0.61*	12.89 ± 1.78
18:1–11	2.26 ± 0.23	1.15 ± 0.13	1.90 ± 0.12	2.03 ± 0.16	2.81 ± 0.23	4.57 ± 1.07*
18:2	20.81 ± 0. 74	20.68 ± 0.71	20.49 ± 0.56	19.94 ± 0.77	20.50 ± 0.41	21.10 ± 0.81
18:3	14.19 ± 1.23	14.98 ± 1.05	14.35 ± 0.53	15.59 ± 0.80	12.30 ± 0.26	14.31 ± 1.47
20:1–11	14.20 ± 0.54	18.88 ± 0.57**	18.23 ± 0.27**	16.85 ± 0.43*	16.20 ± 0.38*	18.64 ± 0.35**
Others	13.91 ± 1.12**	9.14 ± 0.77	9.55 ± 0.43	11.38 ± 0.73*	11.04 ± 0.58*	8.51 ± 1.21
C16–18	72.89 ± 1.72	71.98 ± 1.17	72.23 ± 1.52	71.74 ± 3.19	72.85 ± 1.29	72.81 ± 1.77
SFA	32.06 ± 1.21**	26.95 ± 1.02	23.03 ± 0.73	29.06 ± 0.73*	24.72 ± 1.11	22.17 ± 0.89
UFA	67.94 ± 1.73	73.05 ± 1.58*	76.97 ± 0.82**	70.94 ± 1.13*	75.28 ± 0.71**	77.83 ± 0.56**
UFA/SFA	2.12	2.71	3.34	2.44	3.05	3.51

The values represent means and standard deviation of four independent samples

*FA* fatty acid, *WT* wild type, *KS/kasI kasI* mutant complementation line, *OE* overexpression lines, *UFA* unsaturated fatty acid, *SFA* saturated fatty acid, *US/S* refers to the ratio of unsaturated/saturated FA

The asterisk indicates significant difference in fatty acids composition between transgenic plants and WT (*P < 0.05, **P < 0.01)

### Effect of the KS domain expression on early seedling growth in *Arabidopsis*

**Fig. 7:**
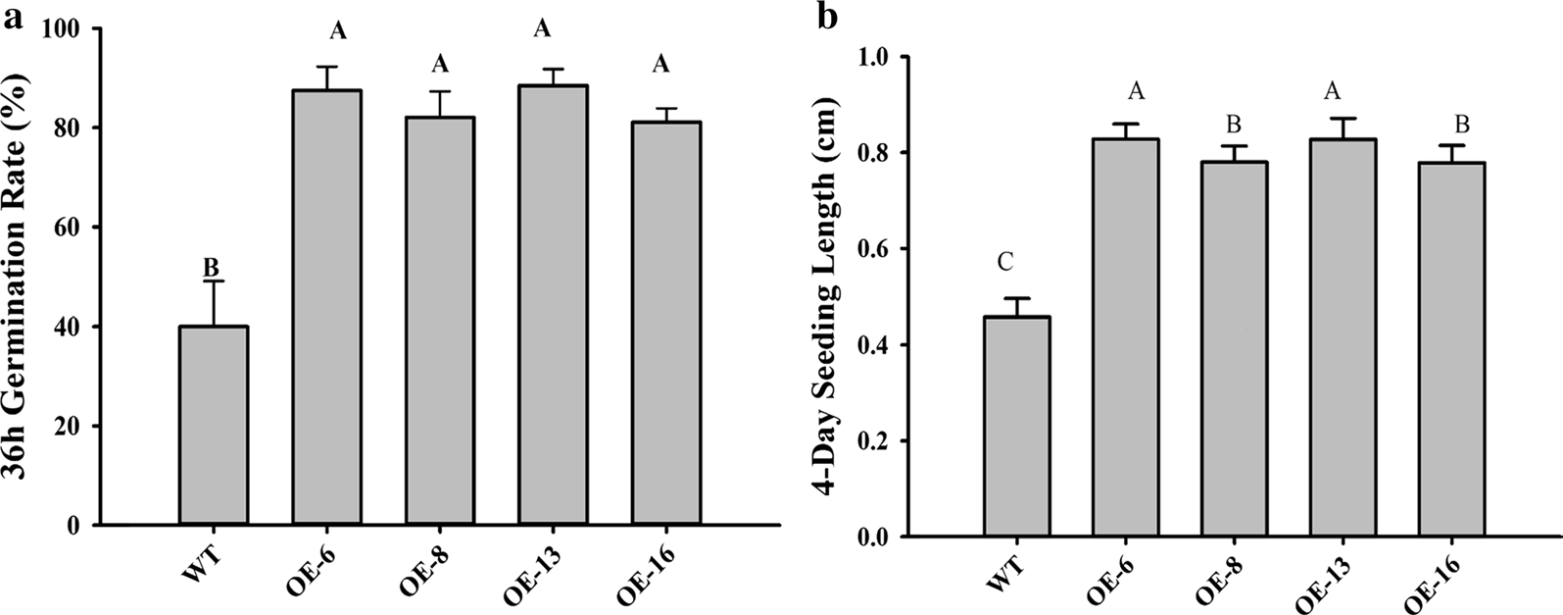
Effect of KS domain expression on seed germination and early seedling growth in *Arabidopsis.* **a** Germination rates of wild type and transgenic lines at 36 h of imbibition. **b** The 4-day seedling length of WT and OE lines. *WT* wild type, *OE* overexpression lines. Values are reported as the means of 10 biological replicates along with standard deviation. The means with the same letters are not statistically significantly different. Statistical analysis of the results was conducted using the one-way analysis of variance (*P* < 0.05)

To examine the growth effects of the KS domain, the growth phenotype of the transgenic *Arabidopsis* plants was carefully observed during the life cycle. No obvious phenotypical difference could be detected between transgenic and untransformed wild-type control except for seed germination and early seedling growth. The seed germination rates of four transgenic lines were considerably higher (217%, 205%, 211% and 203%) than that of the control (Fig. 7a). The lengths of 4-day seedlings of the transgenic lines were all longer compared to the wild type (Fig. 7b). This result indicated the expression of the KS domain from the PUFA synthase in *Arabidopsis* could promote seed germination and seedling growth.

## Discussion

In *Thraustochytrium*, the biosynthesis of VLCPUFAs is catalyzed by a PUFA synthase, a polyketide synthase-like mega-enzyme comprising three subunits each with multiple catalytic domains predicted on the characteristic active site residues [13]. However, the exact functions of these domains in the biosynthesis of these fatty acids are not well characterized. Our previous study indicated that a dissected KS domain from Subunit-B of the PUFA synthase from *Thraustochytrium* could complement the temperature-sensitive phenotype of *E. coli* β-ketoacyl-ACP synthase I (*FabB)* mutant and enhance the total fatty acid production in a wild-type *E. coli* strain [17]. In the present study, this domain was further functionally analyzed in *A. thaliana*. As the *kasI* mutant of *Arabidopsis* was defective in growth and development, and not amenable for re-transformation to express the KS domain for the complementation test, we resorted expressing the KS domain in *Arabidopsis* first and disrupting the endogenous *KASI* genes subsequently using a CRISPR/Cas9 system. Two homozygous insertion mutations of the target gene on the KS-expressed background were identified. Both resulted in the open reading frame shifting, which led to the inactivation of the *KASI* gene [25]. In the two complementation lines, the defective phenotypes of *kasI* knockout mutants such as small seedlings and dwarf plants were not observed anymore (Fig. 3). The RT-PCR analysis showed that transcription of the KS domain was detected in both leaves and developing seeds under the napin promoter, although the transcription level in developing siliques was 10 times higher than that in leaves. The similar expression pattern of the napin promoter has been seen in transgenic *Brassica napus* and tobacco [26, 27]. Nevertheless, the level of the KS domain expression under the napin promoter was enough for the functional complementation in *Arabidopsis*.

After the success of functional complementation, we then overexpressed the KS domain in wild-type *Arabidopsis* under the seed-specific promoter. The result showed that seed weight was increased by 20–40%, and seed oil content was increased by 14% to 28% in four transgenic lines. The degree of the increase was coincided with the expression level of the KS domain in the siliques of these transgenic lines (Fig. 6). In addition, expression of the KS domain also had positive effects in seed germination and early seedling growth. This result suggests that the expression of the KS domain from the PUFA synthase could boost the de novo biosynthesis of fatty acids, thereby increasing the oil content in transgenic seeds.

In plant, the biosynthesis of long-chain fatty acids occurs in plastids by a type II fatty acid synthase complex comprising multiple discrete enzymes [10]. Among these enzymes, three β-ketoacyl-ACP synthases (KASI, KASII and KASIII) are key ones responsible for the condensing reaction, the first of four chemical reactions in a repetitive cycle of fatty acid synthesis by the addition of a two-carbon unit a time donated by malonyl-ACP. KASIII catalyzes the first condensation of fatty acid synthesis between acetyl-CoA/ACP and malonyl-ACP, giving a C4 acyl chain. KASI (β-ketoacyl-ACP synthase I) catalyzes the subsequent condensation reactions from C4 to C16 acyl chains. KASII catalyzes the final condensation of fatty acid synthesis producing a C18 acyl chain. After synthesis, long-chain fatty acids are modified in cytosol and assembled into storage glycerolipid triacylglycerols [28]. Previous studies showed that out of three KAS enzymes, KASI might be the most important in regulating fatty acid biosynthesis in plant. Knocking out/down of *KASI* gene could significantly reduce fatty acid production, while overexpression of it could increase seed weight and total fatty acids in seeds [29, 30]. Here, we showed that overexpression of a KS domain from the PUFA synthase of protist *Thraustochytrium* could increase seed weight by 20–40% and total fatty acids by 48–80% per seed relative to the wild-type control in *Arabidopsis*, which are much higher than those in the overexpression of plant endogenous KASI [30]. The reason for the difference in the two expressions remains to be determined. It is possible that the KS domain protein from the PUFA synthase might simply possess higher condensation activity. In addition, expression of a KS domain of PUFA synthases of microbial origin in plant might own an advantage in minimizing the possible feedback inhibition over the expression of an endogenous KASI [31].

In the past few years, several molecular strategies have been attempted to increase oil production in oilseed crops. One strategy is to increase fatty acid assembly into triacylglycerols by overexpressing acyl-CoA acyltransferase in the Kennedy pathway. For instance, overexpression of lysophosphatidic acid acyltransferase (LPAAT) or DGAT1 significantly increases oil contents in seeds. Fatty acid profile in the oil is determined by substrate specificity of acyl-CoA acyltransferases used for fatty acid acylations [6, 32]. Another strategy is to increase the synthetic efficiency of fatty acids prior to the assembly by overexpression of enzymes directly involved in the biosynthetic process. For instance, overexpression of *ACCase* resulted in an increase in oil content [7, 33–35]. In this strategy, fatty acid profile of the oil would be similar to that of wild type. In the present study, expression of the KS domain from the PUFA synthase significantly enhanced oil content in transgenic lines and at the same time the fatty acid profile of seed oil was also altered to the higher proportion of unsaturated fatty acids. This result indicates the condensation process, particularly the condensation steps from C4 to C16, during the fatty acid biosynthesis might be the limiting step, and overexpression of the KS domain of the *Thraustochytrium* PUFA synthase, although it only shares about 20% amino acid identity to *Arabidopsis* KASI, can increase seed oil and seed mass simultaneously in *Arabidopsis*. The reason why the unsaturated level of fatty acids was also increased in the transgenics remains to be determined. It is probably due to the unique activity of the KS domain of microbial origin. Overexpression of the domain may not only enhance the main condensation reactions in fatty acid synthesis, but also stimulate the activity of downstream fatty acid-modifying enzymes such as acyl-ACP and phospholipid desaturases and FAE-type elongases. Collectively, our research in the enhanced condensation activity by overexpression of the KS domain from protist PUFA synthase resulting in significantly increased seed weight and seed oil offers a new approach to increase oil production in oilseed plants.

## Conclusions

Seed oil is an important renewable bioresource for food and bioproducts. This research provides a new strategy to enhance oil and unsaturated fatty acid production by expressing KS domains from polyketide synthase-like PUFA synthases of microbial origin in oilseed crops for nutritional and industrial uses.

## Methods

### Materials

*Agrobacterium* and *E. coli* media were purchased from Bio Basic Inc. (York, Ontario, Canada). Fatty acids and their standards were obtained from Nu-Chek-Prep, Inc. (Elysian, MN, USA). Q5 DNA polymerase, restriction enzymes, T7 Endonuclease and deoxynucleotide triphosphate (dNTP) were purchased from New England Biolabs (Ipswich, MA, USA). HP Taq DNA polymerase was obtained from Bio Basic Inc. (York, Ontario, Canada). Primers were synthesized from Sigma-Aldrich (St. Louis, MO, USA). GC grade solvents were from Fisher Scientific (Ottawa, ON, Canada). All other chemicals were purchased from Sigma-Aldrich Ltd (Oakville, ON, Canada). The intermediate vector and expression vector for CRISPR/Cas9 system were purchased from Addgene (Watertown, MA, USA).

### Plant materials and growth conditions

Wild-type (Col-0) *Arabidopsis* (*A. thaliana*) seeds were surface-sterilized and remained in darkness for 3 days at 4 °C. Seeds were germinated on a half-strength MS medium [36], and the plates were placed in a growth chamber set to 22 °C under a 16-h-light (120 μEm^−2^ s^−1^)/8-h-dark photoperiod. After 12 days, each seedling was transplanted to pots. Individual plants in each pot were arranged randomly in a tray. When plants began to flower, a 60-cm stick was inserted in each pot to tie the stems, and at maturity, the pot was placed inside an upright rectangular transparent, perforated plastic bag (60 × 6 cm).

### Construction of plant expression vectors

To express the KS domain in *A. thaliana*, two different constructs with different selection and screening marker genes were built. The plastidial overexpression construct contains a KS domain from PUFA synthase (accession no. PRJNA368972) fused with a functional chloroplasts transit peptide (CTP) at the N-terminus [37] and a DsRed2 from *Discosoma sp*. encoding a red fluorescent protein, each under the control of a seed-specific napin promoter [38], as well as an antibiotic kanamycin-resistance gene under the control of a constitutive nos promoter.

For disrupting *Arabidopsis KASI* (*AtKASI*), a CRISPR/Cas9 construct targeting *AtKASI* was built. Two single guide RNAs (sgRNA) targeting *KASI* gene (AT5G46290) were designed using the previous protocol [website: (http://skl.scau.edu.cn)] [39]. The sgRNA sequences were then inserted into an intermediate plasmid behind an AtU3d promoter. The recombinant AtU3d promoter-driving sgRNA cassettes were then inserted into pYLCRISPR/Cas9P_ubi_-B vector [21]. For transgenic seed screening, a napin promoter was used to guide a modified green fluorescent protein (eGFP) gene at the *Spe*I site of pYLCRISPR/Cas9P_ubi_-B.

### Transformation of *Agrobacterium* with recombinant vector

Electrocompetent cells of *Agrobacterium tumefaciens* GV3101 were prepared as follows. *Agrobacterium* cells were grown for 24 h in LB medium with 50 µg/mL gentamicin. When the A_600_ reached 0.7, the cells were chilled on ice and pelleted by centrifugation (5000 rpm for 10 min at 4 °C). The pellet was washed in 1, 0.5 and 0.02 volumes of cold 10% (v/v) sterile glycerol and resuspended in 0.01 volume of cold 10% (v/v) glycerol. The electrocompetent cells were then frozen in liquid N_2_ and stored at ≤ − 70 °C. The *Agrobacterium* cells were transformed by electroporation with 20 to 50 ng of DNA according to the manufacturer’s instructions. Transformed cells were plated on a selective medium (LB agar with corresponding antibiotic), and incubated for 48 h at 28 °C. Transformed cells were grown for 16 h (28 °C, 225 rpm) in 5 mL LB broth with corresponding antibiotic and 50 µg/mL gentamicin. The fidelity of the construct was rechecked by PCR before floral dipping transformation.

### Transformation of *Arabidopsis* and genotyping of transgenics

*Arabidopsis* at the flowering stage grown in the growth chamber was used for transformation using the floral dip method [40]. The transgenic seeds were selected by fluorescence and grown on soil for the next generation. The transgenic plants were genotyped every generation, and the seeds from positive lines were harvested and planted for the next generation. Genomic DNA of wild-type and transgenic plants was extracted with Edward method [41]. The PCR condition was 30 cycles of denaturation at 95 °C for 30 s, annealing at 60 °C for 30 s, and elongation at 72 °C for 30 s with primers F-KASI-1-seq and R-KASI-seq (Additional file 1: Table S1).

### Transcriptional expression analysis

The samples of 7th–8th rosette leaf and developing siliques at 12 to 14 days after flowering (DAF) from T3 overexpressing lines and wild type were collected and frozen in liquid N_2_. RNA was extracted by Qiagen RNeasy Mini Kit (Germantown, MD, USA) and treated with DNase I for 30 min to digest contaminating DNA in samples. The biosynthesis of complementary DNA was carried out using SuperScript™ III Reverse Transcriptase from Invitrogen (Carlsbad, CA, USA). Quantitative RT-PCR was performed with PowerUp™ SYBR™ Green Master Mix from Fisher Scientific (Carlsbad, CA, USA). The PCR conditions were as follows: 50 °C 2 min, 95 °C for 2 min, 40 cycles of 95 °C for 5 s, 60 °C for 1 min, and one cycle of 95 °C for 15 s, 60 °C for 1 min, and 95 °C for 15 s for melt curve stage. The expression of the housekeeping gene *At*Actin-2 (AT3G18780) was used as references [42]. The expression level was normalized by that of Actin-2, and the primer pairs used for the PCR are listed in Additional file 1: Table S1.

### Fatty acid composition and oil content analysis

For fatty acid analysis, single seed was placed in a glass tube with a screw cap and 5 µL of 10 µg/mL heptanoic acid (C17:0) was added as an internal standard for quantification. Two milliliters of 1% (v/v) H_2_SO_4_ in methanol was added to the glass tube containing seed and internal standard. Sample was heated for 10 min at 80 °C, and seed was crushed by glass rod and incubated at 80 °C for 1 h to transmethylate fatty acids into their FAMEs. FAMEs were extracted with 2 mL of hexane and 1 mL of 0.9% NaCl. The total fatty acid profiles and quantity were analyzed on an Agilent 7890A system installed with a DB-23 column (30 m, 0.25 mm, 0.25 µm) [13, 17].

### Seed germination and seedling growth

The surface-sterilized seeds were plated on agar plates of MS medium containing 1% sucrose and incubated in darkness for 3 days at 4 °C. The plates were then placed vertically in a growth chamber set to 22 °C under a 16-h-light (120 μEm^−2^ s^−1^)/8-h-dark photoperiod for 4 days. Then, the germination rate and seedling length were measured.

### CRISPR/Cas9 knockout mutant screening and complementation test

The knockout mutant generated by CRISPR/Cas9 was genotyped in T2 plants by sequencing of target genes. The authentic mutant lines were used for the complementation test. The phenotype of the mutant and mutant complemented lines were observed and recorded every week.

## Additional file

**Additional file 1: Table S1.** Primers used for this study. **Figure S1.** Comparison of sequences surrounding the sgRNA sequences in the *KASI* genes. *KASI*, original *KASI*; *kasI*, *kasI* knockout mutant; *KS*/*kasI*, *kasI* knockout mutant with KS domain; Blue boxes represent PAM sites, and Green boxes represent the target sites. **Figure S2.** Protein content in transgenic seeds overexpressing the KS domain. WT, wild type; OE, overexpression lines. Values are reported as the means of 4 biological replicates along with standard deviation. The means with the same letters are not statistically significantly different. Statistical analysis of the results was conducted using the one-way analysis of variance (*P* < 0.05).

## Data Availability

All data generated or analyzed during this study are included in this article and its Additional file.
