# Supplementary material for: Enhancing oil production in Arabidopsis through expression of a ketoacyl-ACP synthase domain of the PUFA synthase from Thraustochytrium
Source: Biotechnol Biofuels. 2019 Jun 29;12:172. doi: 10.1186/s13068-019-1514-8 (PMC6599236; doi:10.1186/s13068-019-1514-8)
Supplement: Supplementary file 1 — Additional file 1: Table S1. Primers used for this study. Figure S1. Comparison of sequences surrounding the sgRNA sequences in the KASI genes. KASI, original KASI; kasI, kasI knockout mutant; KS/kasI, kasI knockout mutant with KS domain; Blue boxes represent PAM sites, and Green boxes represent the target sites. Figure S2. Protein content in transgenic seeds overexpressing the KS domain. WT, wild type; OE, overexpression lines. Values are reported as the means of 4 biological replicates along with standard deviation. The means with the same letters are not statistically significantly different. Statistical analysis of the results was conducted using the one-way analysis of variance (P < 0.05). [file 13068_2019_1514_MOESM1_ESM.docx]

Submitted to **Biotechnology for Biofuels**

**Manuscript title:** Enhancing oil production in *Arabidopsis* through expression of a ketoacyl-ACP synthase domain of the PUFA synthase from *Thraustochytrium.*

**Authors:** Xi Xie, Dauenpen Meesapyodsuk, Xiao Qiu^*^

**The affiliation** **and address:** Department of Food & Bioproduct Sciences, University of Saskatchewan, Saskatoon, Saskatchewan, Canada

* Corresponding author: Xiao Qiu

Department of Food and Bioproduct Science, University of Saskatchewan, Saskatoon, SK, Canada S7N 5A8

**E-mail address, telephone and fax number:**

Email: [xiao.qiu@usask.ca](mailto:xiao.qiu@usask.ca)

Tel: 1-306-966-2181

Fax: 1-306-966-2181

**Table S1** Primers used for this study

| **Category** | **Primer** | **Sequence (5’ to 3’)** | **Annotation** |
| --- | --- | --- | --- |
| **Expression construct preparation** | F-CTP | ATGGCTTCCTCTATGCTCTCTTCCG | Primers for cloning CTPs |
|  | R-CTP | GAATTCCTTGTCGTCGTCGTCCTTGTAGTC |  |
|  | F-KS-B | GAATTCATGGCCGCGCGCAAC | Primers for cloning KS-CLF domain |
|  | R-KS-L-B | AAGCTTCTACATGTTGTTCTCGCCGCC |  |
| **Genotyping** | KS-B TEST-F | GCACGAGATGCACGATGAGAA | Primers for sequencing and qRT-PCR |
|  | KS-B TEST-R | ATGGCTTTGGTACACGTTGAGG |  |
|  | F-KASI-1-seq | GTGCTTCTCCTCCAAACC | Primers for sequencing |
|  | R- KASI-seq | CAACAAACCCAATAACCAAA |  |
| **Crisper/Cas9 KO**  **construct preparation** | SpeI-Forward | CCGACTAGTTAATGAGATATGCGAGACG | cloning GFP with napin promoter and terminator |
|  | SpeI-Reverse | CCGACTAGTTGTGATTGTATGTGTGTGCT |  |
|  | AtU3d-KASI-F1 | gtcaTCTTCGCAAAGGTCTCGCAT | sgRNA primers |
|  | AtU3d-KASI-F2 | gtcaCGTTTAGGAGCGGAGACAG |  |
|  | AtU3d-KASI-R1 | aaacATGCGAGACCTTTGCGAAGA |  |
|  | AtU3d-KASI-R2 | aaacCTGTCTCCGCTCCTAAACG |  |
| **qRT-PCR** | F-ACTIN2 | GATTCCTGGACCTGCCTCAT | Actin-2 primers for qPCR |
|  | R-ACTIN2 | TACCCGATGGGCAAGTCA |  |
|  | KASI-qPCR-F | AGCCATCGCCACCGTGAA | AtKASI primers for qPCR |
|  | KASI-qPCR-R | CAAGTGGTGGTGATGGATGATG |  |
|  |  | | |


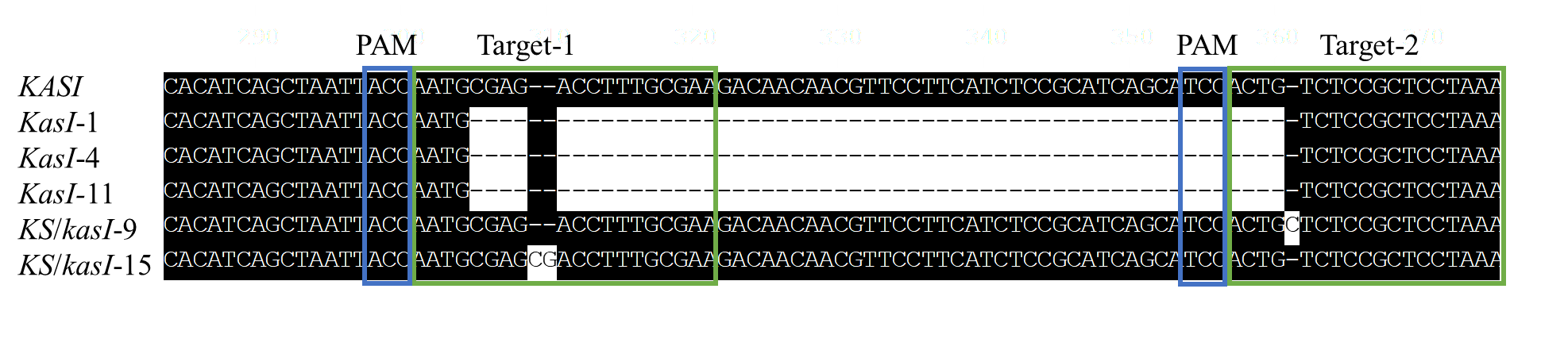
**Fig. S1** Comparison of sequences surrounding the sgRNA sequences in the *KASI* genes. *KASI*, original *KASI*; *KasI*, *kasI* knockout mutant; *KS*/*kasI*, *KasI* knockout mutant with KS domain; Blue boxes represent PAM sites and Green boxes represent the target sites.


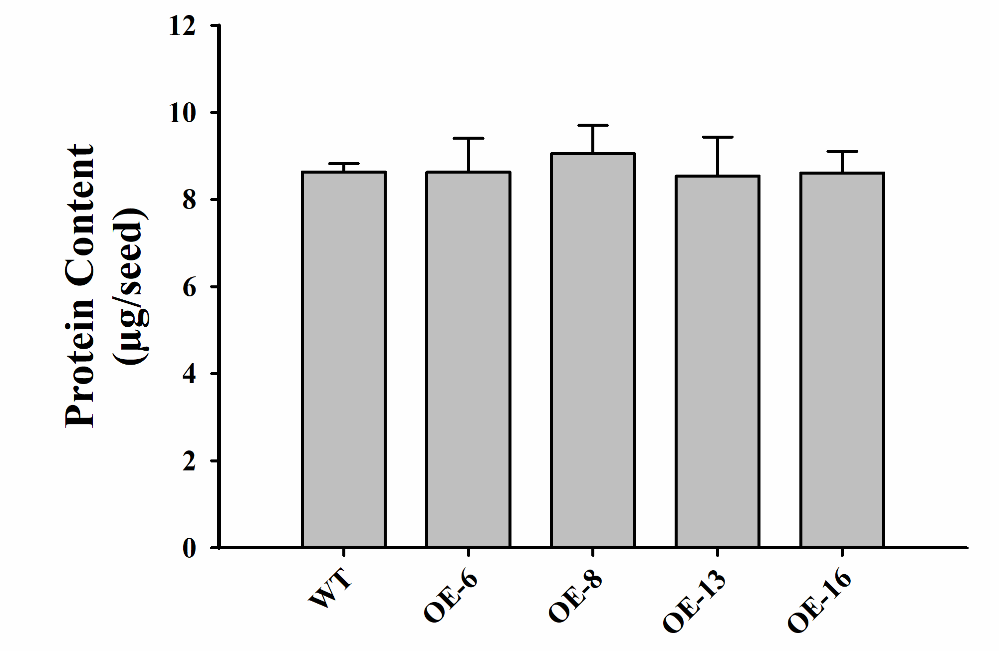

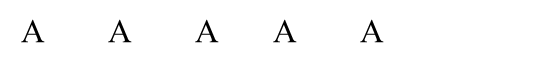


**Fig. S2** Protein content in transgenic seeds overexpressing the KS domain. WT, wild type; OE, overexpression lines. Values are reported as the means of 4 biological replicates along with standard deviation. The means with the same letters are not statistically significantly different. Statistical analysis of the results was conducted using the one-way analysis of variance (P<0.05).
